# Analyzing Patterns in Anesthesiology Residents' Exam Performance Using Data Mining Techniques

**DOI:** 10.5812/aapm-151686

**Published:** 2024-12-07

**Authors:** Maedeh Karimian, Shahabedin Rahmatizadeh, Zeinab Kohzadi, Zahra Kohzadi, Firoozeh Madadi, Ali Dabbagh, DACCPM, Department of Anesthesiology, Critical Care and Pain Medicine

**Affiliations:** 1 Department of Anesthesiology, Anesthesiology Research Center, School of Medicine, Shahid Beheshti University of Medical Sciences,Tehran, Iran; # Collaborators of DACCPM, Department of Anesthesiology, Critical Care and Pain Medicine: Padideh Ansar, Mohsen ArianNik, Sogol Asgari, Moein Daneshmand, Kamal Fani, Alireza Jahangirifard, Fereshteh Kimia, Nastaran Sadat Mahdavi, Kamran Mottaghi, Kobra Rafiei Badi, Hamidreza Samadpour, Parisa Sezari, Marzieh Shahrabi, Alireza Shakeri, Soudeh Tabashi, Ardeshir Tajbakhsh, Mina Vishteh

**Keywords:** Educational Measurement, Artificial Intelligence, Anesthesiology, Internship and Residency, Apriori Algorithm, Data Mining, Education, Artificial Intelligence; Anesthesiology; Internship and Residency; Apriori Algorithm; Data Mining; Education; Medical

## Abstract

**Background:**

Residency is a critical period in the development of medical professionals. It provides hands-on training and exposure to various medical specialties, enabling residents to improve their skills and achieve expertise in their chosen field.

**Objectives:**

This study aimed to extract frequent patterns in annual and board examination performance among anesthesiology residents by analyzing results from the department's weekly exams.

**Methods:**

This cross-sectional study was conducted in the Department of Anesthesiology, Critical Care, and Pain Medicine (DACCPM) from September 2022 to June 2023. Weekly intra-group exams were administered at the university's electronic exam center for residents in their first to fourth years (CA-1 to CA-4), with a total of 61 participants. Learner grades were categorized as excellent (A), good (B), average (C), poor (D), and inferior (E). The Apriori algorithm was employed to extract frequently repeated patterns in these exams and compare them with results from the final national examination.

**Results:**

A total of 24 exams were conducted, with all 61 residents participating. The most frequent patterns, identified with a minimum support of 0.41, revealed that residents generally achieved average scores in exam 7 and very poor scores in exams 1 and 5. The study found a statistically significant relationship between residents’ scores in in-training examinations (ITEs) and their national examination performance.

**Conclusions:**

Analyzing residents’ exam performance using frequent pattern recognition can help identify their strengths and weaknesses. Faculty members can utilize these insights to better plan curricula and enhance the quality of education.

## 1. Background

Residency programs must ensure the quality of training, addressing various aspects of quality definition (1, 2). One of the primary methods to achieve this is by ensuring that trainees meet the required competencies, particularly the six core competencies outlined by the Accreditation Council for Graduate Medical Education (ACGME), including Medical Knowledge as a key component (3-5). The in-training examination (ITE) conducted during residency programs serves as a cornerstone formative tool for enhancing medical knowledge (6, 7). Numerous studies have demonstrated a strong correlation between ITE performance and success in national board exams across various subspecialties. This evidence highlights the critical role of ITE performance as a predictor of future success in the medical profession (8-10).

The growing role of artificial intelligence in medical education, including trainee assessment methods, has been emphasized (11). However, further research is necessary to identify the most effective AI tools (12).

This study adopted a novel approach by employing artificial intelligence to analyze residents' study patterns. Additionally, it evaluated which aspects of reference materials were most influential in improving residents' performance on both annual and national board exams.

We applied the Apriori algorithm to analyze our results. This algorithm utilizes prior knowledge to make predictions and decisions without the need for new data. It is commonly used in machine learning and data analytics to identify and classify data based on existing patterns. The Apriori algorithm effectively extracts insights from datasets, enabling the mining of item sets and the generation of actionable association rules (13, 14).

## 2. Objectives

This study was designed to extract frequent patterns in annual and board examinations for the anesthesiology residents in the Department of Anesthesiology, Critical Care, and Pain Medicine (DACCPM) at Shahid Beheshti University of Medical Sciences (SBMU), Tehran, Iran.

## 3. Methods

### 3.1. Study Design, Objectives, Setting, and Participants

This cross-sectional study, conducted from September 2022 to June 2023, focused on the anesthesiology residents at Shahid Beheshti University of Medical Sciences (SBMU) in Tehran, Iran. The Department of Anesthesiology, Critical Care, and Pain Medicine (DACCPM) implemented weekly in-training examination (ITE) assessments at the electronic exam center, ensuring that all clinical anesthesiology residents, from first year (CA-1) to fourth year (CA-4), received regular evaluations.

The objective of the study was to extract frequent patterns in the annual and board examinations for the anesthesiology residents in the DACCPM, SBMU, including:

- The possible relationship between the frequency of the ITEs and the success rate in the annual exam and the national board exam.

- Identification of the exam content most important in predicting success, i.e., determining which parts of the exam book references should be prioritized to improve the success rate.

- The effect of exam seasons on the success rate.

- Whether the content of the exam plays a role as a potential predictor of the success rate.

Our study involved a sample size of 61 dedicated residents, emphasizing their commitment to education and professional development.

To ensure the taxonomy of the exam questions aligned with the taxonomy of the board exam questions, we used the blueprint of the national board exam as the original template. We then divided the “question taxonomy model” across all the exams based on the individual chapters in each exam. A strict adherence to the blueprint was maintained, resulting in nearly full alignment with the national board exam blueprint.

### 3.2. Ethical Approval

We are pleased to announce that this study has been formally approved as a research project by the Deputy of Research at Shahid Beheshti University of Medical Sciences in Tehran, Iran. Ethical guidelines were rigorously followed, as demonstrated by the ethics approval code from the Research Ethics Committees of the Vice-Chancellor for Research Affairs at Shahid Beheshti University of Medical Sciences: IR.SBMU.RETECH.REC.1402.413. This approval affirms our commitment to ethical research standards. To maintain ethical standards, participant confidentiality was ensured, and data were analyzed only after being anonymized.

### 3.3. Content of the In-training Examination and National Board Exams

Under the expert guidance of the National Board of Anesthesiology, we have identified essential textbooks as exclusive references for optimal study and exam preparation:

1. Miller's Anesthesia, 9th Edition, 2020; Editors: Michael A. Gropper, Lars I. Eriksson, Lee A. Fleisher, Jeanine P. Wiener-Kronish, Neal H. Cohen, Kate Leslie. Elsevier Publications (This cornerstone reference comprises 70% of all test questions, providing a comprehensive foundation for our knowledge.).

2. Stoelting's Anesthesia and Co-Existing Disease, 8th Edition, 2021; Editors: Roberta L. Hines, Stephanie B. Jones. Elsevier Publications (This pivotal ancillary textbook contributes 15% of all test questions, offering crucial insights into coexisting conditions.).

3. Textbook of Critical Care, 8th Edition, 2022; Editors: Jean-Louis Vincent, Frederick A. Moore, Rinaldo Bellomo, John J. Marini. Elsevier Publications (This complementary reference accounts for 15% of the test questions, enhancing our understanding of critical care scenarios.).

In total, 24 ITEs have been conducted, with content and references outlined in Table 1. Each exam consists of 20 selected-response questions, including multiple-choice, matching, and true/false formats, promoting a rigorous assessment environment. The national ITE includes 150 multiple-choice questions that are meticulously aligned with these references.

**Table 1.** The In-training Examination Description

Classification of the Residents	Min-support	Generated Pattern
**Total residents**	0.41	(23), {ITEs 1 and 5: D Score}
**Residents scoring A in the final ITE**	0.40	(24 17, 20: A Score), {ITE 13: C Score}, (24: D Score), {ITE 6: E Score}
**Residents scoring B in the final ITE**	0.43	{ITEs 2,7: C Score}, {ITEs 1, 5: D Score}
**Residents scoring C in the final ITE**	0.42	{ITEs 3, 15, 16, 17, 21: C Score}, {ITEs 1, 5, 11: D Score}, {ITE 13: E Score}
**Total CA-1 residents**	0.42	{ITEs 9, 19, 20: A Score}, {ITE 7: C Score}, {ITEs 1,2,4,5,18, 21, 24: D Score}, {ITE 6: E Score}
**CA-1 residents scoring A in the final ITE**	0.42	{ITEs 9, 15, 16, 17, 19, 20: A Score}, {ITE 22: B Score}, {ITEs 7, 13,18: C Score}, {ITEs 1,2,4,5,9, 21, 24: D Score} {ITE 6: E Score}
**CA-1 residents scoring B in the final ITE**	0.40	{ITE 9: A Score}, {ITE 22: B Score}, {ITEs 2, 7; C Score}, {ITEs 1, 4,5, 11,18: D Score}, {ITEs 1, 5, 11: D Score}, {ITEs 6, 24: E Score}
**CA-2 residents scoring B in the final ITE**	0.40	{ITE 20: B Score}, {ITEs 2, 12, 13,16, 17: C Score}, {ITEs 1,3, 4,9,11,18,21: D Score}, {ITEs 5,14,19,22, 24: E Score}
**CA-3 residents scoring B in the final ITE**	0.42	{ITEs 16, 17: A Score}, {ITE 13: B Score}, {ITEs 3,7, 12,18: C Score}, {ITEs 1, 5, 10, 11: D Score}, {ITE 10: E Score}
**Total CA-4 residents**	0.41	{ITEs 16, 17, 20: B Score}, {ITEs 5, 9, 14,16,22: C Score}, {ITE 2: D Score}, {ITE 10: E Score}
**CA-4 residents scoring B in the final ITE**	0.44	{ITEs 3, 11, 15: A Score}, {ITEs 6,16, 17,20,22: B Score}, {ITEs 5, 7, 9, 14, 22: C Score}, {ITE 10: E Score}
**CA-4 residents scoring C in the final ITE**	0.50	{ITE 6: B Score}, {ITEs 15, 16, 17, 21, 22: C Score}, {ITEs 5, 11,18: D Score}, {ITEs 10,13: E Score}

Abbreviation: ITE, in-training examination.

We aim to develop an algorithm that accurately predicts final grades on the national ITEs, utilizing the results from formative ITEs as essential evaluation tools. By using the National Anesthesiology Exam blueprint as our foundational template, we ensure that the ITE content is thoughtfully designed to reflect this national standard. This strategic approach enables us to excel in both preparation and assessments.

### 3.4. Inclusion Criteria

All anesthesiology residents at Shahid Beheshti University of Medical Sciences from September 2022 to June 2023 are encouraged to participate, provided they give informed consent.

### 3.5. Exclusion Criteria

Anesthesiology residents who were unable to participate in the national ITE due to reasons such as resignation or illness will be excluded from the study.

### 3.6. Data Preprocessing

The grades were categorized into five distinct groups: excellent (A), good (B), average (C), poor (D), and inferior (E) (Table 2). Subsequently, we employed the one-hot encoding method to convert these categories into a binary format. One-hot encoding is a powerful technique that makes categorical data accessible for machine learning models. It transforms each entry of a categorical variable into a vector of zeros and ones, ensuring that only one element is set to one (1), while all others remain zero (0). By utilizing this method, machine learning models can effectively process categorical data, uncovering and learning valuable patterns inherent in the information (15).

**Table 2.** Classification of In-Training Examination Scores

Category of ITE Scores	Academic Year			
	1	2	3	4
	E, D, C, B, A	E, D, C, B, A	E, D, C, B, A	E, D, C, B, A
**ITE 1**	0,7,10,12,14,20	0,9,11,13,16,20	0,10,13,15,17,20	0,11,14,16,18,20
**ITE 2**	0,8,10,12,14,20	0,10,13,15,16,20	0,11,14,16,17,20	0,12,15,16,18,20
**ITE 3**	0,8,10,12,14,19	0,10,13,15,16,19	0,10,13,15,17,19	0,11,13,16,18,19
**ITE 4**	0,7,10,12,15,20	0,9,11,13,16,20	0,10,12,14,17,20	0,11,13,15,18,20
**ITE 5**	0,8,11,13,15,23	0,9,13,15,16,23	0,10,14,16,20,23	0,11,15,18,21,23
**ITE 6**	0,9,11,13,15,20	0,11,13,15,16,20	0,12,14,15,17,20	0,11,14,16,18,20
**ITE 7**	0,7,10,12,14,20	0,8,11,13,15,20	0,10,12,14,16,20	0,11,13,16,18,20
**ITE 8**	0,8,10,13,15,21	0,9,11,14,16,21	0,10,12,15,17,21	0,11,13,16,18,21
**ITE 9**	0,9,11,13,15,20	0,10,12,14,16,20	0,11,13,15,17,20	0,12,14,16,18,20
**ITE 10**	0,10,13,15,20,26	0,13,17,19,22,26	0,14,18,20,23,26	0,15,19,21,24,26
**ITE 11**	0,9,11,13,15,20	0,10,13,15,16,20	0,11,14,15,17,20	0,12,14,16,18,20
**ITE 12**	0,8,10,12,14,22	0,9,11,13,15,22	0,10,12,14,17,22	0,11,13,16,19,22
**ITE 13**	0,8,10,12,14,21	0,9,11,13,15,21	0,10,12,14,17,21	0,11,13,16,19,21
**ITE 14**	0,9,11,13,16,24	0,10,13,16,17,24	0,11,14,17,20,24	0,12,15,18,21,24
**ITE 15**	0,8,10,12,14,20	0,9,11,13,15,20	0,10,12,14,17,20	0,11,13,16,18,20
**ITEs 16 and 17**	0,8,10,12,14,20	0,9,11,14,16,20	0,10,12,15,17,20	0,11,13,16,18,20
**ITE 18**	0,8,10,12,14,20	0,9,11,13,15,20	0,10,12,14,16,20	0,11,13,16,18,20
**ITE 19**	0,8,10,12,14,20	0,9,11,13,15,20	0,10,12,15,16,20	0,11,13,16,18,20
**ITE 20**	0,8,10,12,14,21	0,9,11,13,15,21	0,10,12,14,17,21	0,11,13,16,19,21
**ITE 21**	0,7,10,13,15,21	0,10,12,14,16,21	0,11,13,14,17,21	0,12,14,16,19,21
**ITE 22**	0,7,10,12,15,20	0,10,12,14,16,20	0,11,13,15,17,20	0,11,13,16,18,20
**ITE 23**	0,7,10,12,16,20	0,10,12,15,17,20	0,11,13,16,18,20	0,12,14,17,19,20
**ITE 24**	0,25,35,45,55,80	0,30,40,50,60,80	0,35,45,55,65,80	0,40,50,60,70,80
**Final ITE**	0,65,75,95,134	0,75,85,105,134	0,85,95,115,134	0,95,105,125,134

Abbreviation: ITE, in-training examination.

### 3.7. Selection of Data Mining Algorithm

To create a highly effective prediction model for students' test scores, we rigorously analyzed five powerful data mining techniques: Naive Bayes, Decision Trees, K-Nearest Neighbors, Artificial Neural Networks, and Support Vector Machines. Each method offers unique strengths that can significantly enhance our understanding of student performance (16).

The Apriori algorithm stands out as a powerful tool for extracting valuable patterns from data. It effectively mines item sets and generates association rules, enabling a comprehensive analysis of relational databases in our study. By leveraging prior knowledge or assumptions, Apriori allows us to make informed predictions and decisions without relying on new data. This algorithm is widely recognized in machine learning and data analytics for its ability to predict and classify data based on established patterns and relationships (14, 17-19).

As a formidable unsupervised machine learning resource, the Apriori algorithm excels in uncovering meaningful association rules, making it vital for effective data-driven decision-making. Our approach focuses on identifying critical items within the ITE scores database, systematically expanding them into larger sets to ensure comprehensive data capture. The frequent item sets generated through this algorithm provide a solid foundation for developing insightful association rules, allowing us to uncover significant trends and deepen our understanding of the data. Embracing the Apriori algorithm can greatly enhance our analytical capabilities and drive impactful results (17-20).

### 3.8. Evaluation

To effectively evaluate the algorithm, we employed the Min-Support metric, a crucial standard in pattern mining that determines the minimum frequency required for an item to be considered frequent. This criterion ensures that a pattern is supported by a substantial amount of data from the dataset, validating its significance. By leveraging this criterion, we successfully uncovered meaningful and reliable patterns within the ITE scores dataset, underscoring its importance in our analysis.

### 3.9. Potential Sources of Bias

There are three potential sources of bias in the study, which we describe briefly below (21, 22):

- Content relevance: To address bias effectively, we meticulously ensured that our test content covered a range of topics, closely adhering to the national board's comprehensive blueprint.

- Cultural sensitivity: To eliminate bias, we are committed to providing a uniform examination environment, ensuring fairness for every student involved. This commitment should reassure you of the fairness of our process.

- Stereotyping: To combat bias, we crafted innovative and original questions that promote critical thinking, thereby enhancing the exam experience. This approach aims to engage students more effectively in the examination process.

## 4. Results

Among anesthesiology residents, 61 individuals met the inclusion criteria, and 24 ITEs were conducted before the final ITE. Table 2 shows the classification of all ITEs according to academic year (first: CA-1, second: CA-2, third: CA-3, and fourth: CA-4) in five categories: very good (A), good (B), average (C), poor (D), and very poor (E).

At the CA-2 and CA-3 levels, most residents' final ITE scores were categorized as "B." Therefore, the most frequent patterns were calculated for this category, but could not be calculated for the other categories. At the CA-4 level, the final ITE scores for most residents were in categories B and C. Frequent patterns were observed in 41% of all residents, specifically in the E score in ITE 7 and D scores in ITE 1 and ITE 5. The most frequent patterns (Min-Support: 50%) were associated with CA-4 residents who scored C in the final ITE. More details of the most frequent ITE score patterns are provided in Table 3. The demographic characteristics of the participants are presented in Table 4. 

**Table 3.** The Most Frequent Pattern Extracted in ITE, In-training Examination for Residents

ITE Number	ITE Description
**ITE 1**	Lung physiology and respiration Miller 2020, Co-Exist 2022
**ITE 2**	Blocking and neuromuscular drugs Miller 2020
**ITE 3**	Anesthesia in transplantation Anesthesia in organ transplantation, brain death, old age and burns Miller 2020
**ITE 4**	Cardiac Physiology and Arrhythmia Miller 2020
**ITE 5**	conduction disorders, valvular disorders, heart failure and ischemic heart disease Co-Exist 2022
**ITE 6**	Pre-op review, familiarization with co-morbidities in anesthesia and CPR Miller 2020
**ITE 7**	Anesthesia in adult and pediatric heart surgery Miller 2020
**ITE 8**	Getting to know inhalation agents Miller 2020
**ITE 9**	Consciousness, memory, sleep medicine, immune diseases and psychiatry Miller 2020, Co-Exist 2022
**ITE 10**	Intravenous agents, airway management and workplace safety Miller 2020
**ITE 11**	Management of acute and chronic pain, anesthesia in special conditions and palliative medicine Miller 2020
**ITE 12**	Familiarity with narcotics, non-narcotic and herbal drugs and anesthesia management in bariatric, orthopedic and eye surgeries Miller 2020, Co-Exist 2022
**ITE 13**	Neuroanesthesia Miller 2020
**ITE 14**	Management of patient position in anesthesia, anesthesia in ENT and familiarization with diseases affecting peripheral and central nerves Miller 2020, Co-Exist 2022
**ITE 15**	Anesthesia management in gynecological, fetal and outpatient surgery Miller 2020, Co-Exist 2022
**ITE 16,17**	Physiology of digestion and liver and anesthesia in children, Anesthesia management in vascular surgeries, cardiac trauma, hematological and skin diseases Miller 2020, Co-Exist 2022
**ITE 18**	Familiarity with local anesthetics and regional anesthesia for adults and children Miller 2020
**ITE 19**	Anesthesia in trauma and prehospital and PICU Miller 2020
**ITE 20**	Anesthesia in kidney and urology surgeries Miller 2020, Co-Exist 2022
**ITE 21**	Patient safety and transfusion and management of critically ill patients Miller 2020, Co-Exist 2022
**ITE 22**	Management of congenital heart diseases, cancer, chronic pain and old age Co-Exist 2022
**ITE 23**	Procedures and management of pulmonary diseases in special department Dellinger 2019
**ITE 24**	Cardiovascular diseases, special section Dellinger 2019

Abbreviation: ITE, in-training examination.

**Table 4.** Demographic Characteristics of Participants (n = 61) ^a^

Variables	Values
**Gender**	
Female	40 (65.57)
Male	21 (34.43)
**Mean age**	34.39 ± 4.68
**Marital status **	
Single	29 (47.54)
Married	32 (52.46)
**GP university brigade**	
Type 1	22 (36.06)
Type 2	37 (60.66)
Type 3	2 (3.28)
**Academic year**	
First year	20 (32.79)
Second year	10 (16.39)
Third year	14 (22.95)
Fourth year	17 (27.87)

^a^ Values are expressed as mean ± SD or No. (%).

## 5. Discussion

The results of the current study demonstrate a promising relationship between the frequency of ITE and success rates in both the annual exam and the national board exam. The study identifies specific content areas within the exam materials that are crucial for predicting success, providing a roadmap for exam preparation and performance improvement. Additionally, certain exam topics emerged as significant predictors of success, offering further support for targeted preparation. However, the effect of exam seasons on success rates remains uncertain. Using advanced data mining techniques, we uncovered critical patterns in the Annual Examinations of Anesthesiology Residents, a discovery that holds great potential for enhancing exam preparation strategies.

The results of the current study also demonstrated a statistically significant relationship between residents' scores in ITEs and their final scores in both the annual and national board exams. Previous studies have shown that ITEs are an efficient method for improving resident outcomes in important exams (6, 23-25). Our findings are consistent with another study that demonstrated a significant correlation between the USMLE Step 1 score and performance on both the American Board of Anesthesiology (ABA) residency ITE and the traditional certification examination among anesthesiology residents (26-28).

The connection between specific content areas in the exam materials is crucial for predicting success. Our study identified which topics should receive more attention and focus during preparation. Additionally, we provided a roadmap for improving exam performance. Some exam topics emerged as strong predictors of success rates, offering encouragement for targeted study efforts. The patterns observed in the exams also highlighted gaps in teaching, exam structure, and residents' knowledge.

Furthermore, we pinpointed the critical exam topics that are vital in predicting success rates. Emphasizing these specific areas in the exam reference materials can significantly enhance outcomes. Our analysis also revealed which chapters of the exam content are most likely to influence success rates, allowing us to target our efforts effectively. However, further exploration is needed to understand how different exam seasons might impact these rates, which represents an opportunity for future investigation.

The results of our study, in conjunction with previous global studies, demonstrate that the connection between frequent patterns and final exam outcomes must be explicitly aligned with educational objectives.

The recurring pattern among all residents was that poor scores were obtained in ITE 1 and ITE 5, indicating that these two tests had issues with the design and structure of the questions. As a result, a meeting with the test committee was held, and a detailed examination of each question was conducted. For questions identified as problematic, feedback was provided to the faculty members responsible for designing them. Additionally, based on previous experience in the DACCPM (29, 30), a training workshop was organized for faculty members to improve the design of standard clinical questions.

Among the residents who received a very good grade (A) in the annual national exam, the most common pattern was obtaining a very poor grade in ITE 6. Since residents who received good or average grades in the end-of-year promotion test did not perform poorly in ITE 6, it is possible that this ITE did not have an effective role in the annual national exam. As a result, it was reviewed by the DACCPM examination committee, which found that the chapters on pre-operative evaluation, co-morbidities in anesthesia, and CPR did not carry significant weight in the annual national exam. Therefore, it was decided to merge these topics with others in the following year and to stop examining them separately. On the other hand, given that these residents performed poorly in this test, it was decided that these topics should receive much more attention in the following year, especially for the CA-1 residents.

Additionally, residents who had excellent results in the annual ITE exam also performed very well in ITEs 15, 16, and 17. Conversely, residents who scored average marks in the annual ITE exam had average performance in these three ITEs. This finding suggests that the topics covered in these three ITEs played an important role in the annual ITE, as residents who excelled in these topics were able to achieve better final scores. Therefore, this issue was raised and discussed in the “Assessment, Evaluation, and Exams Subcommittee,” leading to the decision to address the following chapters more specifically in upcoming ITEs, ensuring that all residents could improve in these areas:

- OBGYN anesthesia

- Anesthesia management in fetal surgeries

- Anesthesia in outpatient settings

- Physiology of the GI Tract and liver

- Pediatric and neonatal anesthesia

- Anesthesia for vascular surgeries

- Anesthesia for cardiac trauma

- Anesthesia for patients with underlying hematologic and dermatologic disorders

It could also be suggested that these topics should be covered in 5 ITEs instead of only 3 ITEs, to provide more comprehensive assessments and to enhance the knowledge base and competency practice on these topics.

The results of the frequent patterns among residents with “good final scores” and “very good final scores” in the final annual ITE demonstrated that, in both groups, the score for ITE 2 was not at the desired level. It seems that the topics covered in this test were not sufficiently addressed in the final ITE. The “Assessment, Evaluation, and Exams Subcommittee” of the DACCPM reviewed this finding and, recognizing the importance of these topics, decided not to remove or reduce them. Instead, the subcommittee decided to integrate the relevant topics into other exams.

The findings of the current research revealed a recurring pattern among all residents whose final scores in the ITE were average (C) in ITE 13; they performed very poorly. As a result, it was decided to improve the quality of teaching for these topics in the next year, and to increase the number of questions on this topic to ensure comprehensive coverage of the content in a more favorable way.

On the other hand, since the CA-4 (final year) residents performed very poorly in this exam, with an average improvement grade (C), this group of residents underwent a re-evaluation process, along with a special and intensive supplementary training course. Similarly, in ITE 11, all residents who performed poorly in the final annual ITE exam (with an average grade of C) were reviewed, and the “Assessment, Evaluation, and Exams Subcommittee” of the DACCPM approved that the subject of this exam should be taught exclusively and additionally in three-month workshops for all residents.

The findings of the research showed that all the final-year residents performed very poorly in ITE 10. Since other residents performed better in this exam, this issue was specifically investigated by the “Assessment, Evaluation, and Exams Subcommittee” of the DACCPM. It was proposed that the educational challenges during the COVID era (31) were one of the main causes of this problem. As a result, it was decided to hold a one-month intensive training course for these residents before graduation, which would include:

- Intravenous agents

- Airway management

- Environmental safety

Based on these findings, the number of ITEs had a positive effect on achieving better results in the final exam. However, in planning for the next academic years, the effect of exam seasons on the final exam should be considered, and balanced planning should be made. Additionally, those ITEs that proved effective and those in which the CA residents did not perform well should be addressed more carefully for further assessment.

The results of the current study demonstrated that the relationship between residents' scores in ITEs and their final scores in the National Board exam was statistically significant. As shown in previous studies, ITEs are an efficient method to improve residents' outcomes in major exams (6, 23-25). Our results were supported by another study that demonstrated a significant correlation between USMLE Step 1 scores and performance on both the American Board of Anesthesiology (ABA) residency ITE and the traditional certification examination among anesthesiology residents (26-28).

Additionally, a previous systematic review found that passing board examinations was significantly associated with strong performance on ITEs among various specialists (8). In this study, higher scores on certain ITEs were associated with higher final exam scores. However, there have been relatively few studies assessing the role of an algorithmic approach in predicting the final success rate, which is a novel finding in our study (32, 33).

In general, most students scored poorly on the initial exams; however, better scores were achieved as the number of exams increased (34-36). The fact that being in an exam situation helps residents become more accustomed to the process cannot be ignored. Although this program covered all the chapters from three reference books, determining the optimal number of ITEs will require further study and investigation.

### 5.1. Conclusions

The primary focus of this study was to thoroughly evaluate the structure and consistency of the ITEs used to assess anesthesiology residents at SBMU. We aimed to determine their effectiveness in preparing candidates for the annual national exam and the national board exam. Our analysis focused on how well the ITEs align with the standards of these exams and whether residents' performance on the ITEs provides a reliable measure of their future success on both the annual national exam and the national board exam. This evaluation is crucial for ensuring that our training methods are effective and meet professional expectations.

Additionally, we identified the exam topics most important for predicting success rates, highlighting specific areas in the exam reference materials that should receive greater emphasis to improve outcomes. We also determined, to some extent, the contents of the exam as potential predicting factors for success; that is, we outlined the chapters most likely to affect the success rate. However, the potential impact of exam seasons on the success rate still needs further investigation.

Based on these findings, the number of ITEs was shown to positively affect better results in the final exam. However, when planning for future academic years, the effect of exam seasons on the final exam should be considered, and balanced planning should be made for both the number and timing of exams. Those ITEs that have been effective, as well as those in which the CA residents did not perform well, should be reviewed in a more detailed manner for further assessment.

Our results employed a unique approach: artificial intelligence identified residents' study patterns. Additionally, we discovered which aspects of the reference books significantly contributed to the residents' success rates in their final scores for both the annual and national board exams.

### 5.2. Limitations

1. Drawing conclusions about the effect of the number of exams on board exam scores based solely on data from one academic year may not be sufficient. To establish a more robust understanding, it would be helpful to include data from another academic year with a lower number of exams. This study served as the baseline, or pilot, for a larger study. We have planned to continue this process in more comprehensive future studies.

2. We could not conclusively determine which topics are deemed important for the board exam based solely on the scores and their relationships. A more thorough analysis could strengthen our claims in this area, which will be addressed in a future study.

3. The moods and personal circumstances of each resident, as well as any challenges faced throughout the year or before each test, undeniably influence our results. Unfortunately, the limitations of our study prevented us from thoroughly assessing and accounting for these critical factors.

4. The individual personal data of each resident could greatly influence our findings. However, we did not collect this data, and it is essential to conduct further investigations to understand its impact on the study results in future research. We also compared each resident's final performance on the annual or board exam to their performance in group exams. While the absence of this data may be a limitation, it is a critical aspect of our study that cannot be overlooked.

5. The findings of this study were based on ITEs from our department, although several colleagues from other departments participated. If the study had included a broader range of external evaluators, it would have greatly enhanced the external validity of the results, making them more applicable to other contexts.

## Data Availability

Data will be made available upon request.
